# Treatment of capecitabine-induced hand-foot syndrome using a topical retinoid: A case report

**DOI:** 10.3892/ol.2013.1706

**Published:** 2013-11-26

**Authors:** MASAFUMI INOKUCHI, SATOKO ISHIKAWA, HIROYUKI FURUKAWA, HIROYUKI TAKAMURA, ITASU NINOMIYA, HIROHISA KITAGAWA, SACHIO FUSHIDA, TAKASHI FUJIMURA, TETSUO OHTA

**Affiliations:** Department of Breast Oncology, Division of Cancer Medicine, Graduate School of Medical Science, Kanazawa University, Kanazawa, Ishikawa 920-8641, Japan

**Keywords:** retinoid, adapalene, hand-foot syndrome, capecitabine, heparin-binding epidermal growth factor-like growth factor, chemotherapeutic resistance

## Abstract

Capecitabine is a chemotherapeutic drug used in patients with breast, colon and gastric cancer. Hand-foot syndrome (HFS) is a type of dermatitis that frequently occurs as a reaction to capecitabine. To date, no effective strategies have been found to prevent or reverse HFS. Furthermore, chemotherapy induces an elevation in the expression of heparin-binding epidermal growth factor-like growth factor (HB-EGF), and this activation represents a critical mechanism for the induction of chemotherapeutic resistance. Adapalene is a third-generation synthetic retinoid. Topical retinoids are important therapeutic anti-aging agents for managing photodamaged skin, and are known to increase HB-EGF levels, which is important for skin wound healing. Accordingly, the current report focused on the topical retinoids that increase HB-EGF expression in the skin, and we hypothesized that these topical retinoids induce local chemotherapeutic resistance in the skin of patients receiving chemotherapy and consequently, decrease the cutaneous side-effects of chemotherapy. This report presents a case of the successful treatment of refractory HFS induced by capecitabine using the topical application of adapalene. Topical adapalene was applied for 3 months and significantly reduced inflammation and pain following chemotherapy. Topical retinoids may have the potential to effectively treat capecitabine-induced HFS by increasing HB-EGF expression and decreasing cutaneous side-effects. Further studies are required to establish the therapeutic efficacy of topical retinoids on HFS.

## Introduction

Capecitabine is a novel oral fluoropyrimidine carbamate rationally designed to allow selective 5-fluorouracil (5-FU) activation in tumor tissues (1). Capecitabine is metabolized to 5-FU within tumors and produces certain side-effects characteristic to 5-FU. Although capecitabine is generally well tolerated, hand-foot syndrome (HFS) is the most common clinical adverse reaction. HFS, also called palmar-plantar erythrodysesthesia, is a distinctive and relatively frequent dermatological toxic reaction associated with certain chemotherapeutic agents, specifically capecitabine, infusional fluorouracil and liposomal doxorubicin (2). The manifestations of HFS have been classified into 3 grades according to their severity (3). HFS (of all grades) occurred in ~50% of patients in the early phase II studies of capecitabine as a single-agent therapy for metastatic breast and colorectal cancers, with 10% of patients experiencing severe HFS (grade 3) (3).

The pharmacological mechanism of capecitabine-associated HFS remains unclear. The elimination of the eccrine gland system by capecitabine may result in HFS. Asgari *et al*(4) previously indicated that capecitabine affects the eccrine system due to increased levels of thymidine phosphorylase in the skin keratocytes, leading to capecitabine metabolite accumulation. Furthermore, Mrozek-Orlowski *et al*(5) previously hypothesized that this cytotoxic drug may be excreted in sweat, making the palms and soles more prone to HFS. This is due to the large number of eccrine sweat glands in these extremities, as areas that contain a high concentration of apocrine sweat glands are affected. Vascularization and increased pressure and temperature in the hands and feet may perpetuate this effect (3).

Furthermore, previous studies have indicated that heparin-binding epidermal growth factor-like growth factor (HB-EGF) is also associated with chemotherapeutic resistance. Drug resistance is one of the principal reasons for the failure of chemotherapy. Chemotherapy also induces the elevated expression of HB-EGF, and HB-EGF activation represents a critical mechanism for the induction of chemotherapeutic resistance (6).

Topical retinoids are important therapeutic anti-aging agents for the management of photodamaged skin. Adapalene is a third-generation synthetic retinoid and a naphthoic acid derivative that has specific pharmacological activities similar to regular retinoids (7). Adapalene also demonstrates a low potential for irritation and a direct anti-inflammatory effect. Topical retinoids markedly increase HB-EGF expression in human keratinocytes (8) and are considered to be important for skin wound healing (9).

Therefore, the current report focused on the topical retinoids that increase HB-EGF expression in the skin. We hypothesized that topical retinoids induce local chemotherapeutic resistance in the skin of patients receiving chemotherapy and consequently, decrease the cutaneous side-effects of chemotherapy.

Furthermore, it was considered that HFS may be responsive to adapalene. This report presents a case of the successful treatment of refractory HFS induced by capecitabine using the topical application of adapalene. Written informed consent was obtained from the patient.

## Case report

A 75-year-old female was diagnosed with invasive ductal carcinoma without metastasis (pT2N0M0). The patient was treated with two courses of chemotherapy consisting of docetaxel plus trastuzumab for one year, as the tumor cells were weakly positive for estrogen receptors and positive for human epidermal growth factor receptor type 2 (HER2). Following completion of the chemotherapy, tamoxifen was administered. One year after the initial surgery, axillary lymph node metastasis was detected and the patient underwent axillary lymph node clearance surgery. The patient was diagnosed with pleural metastasis ~5 months after the second surgery, and oral capecitabine was initiated [capecitabine (Xeloda) chemotherapy, at a daily dosage of 1,657 mg/m^2^ in divided doses]. Each cycle of therapy consisted of three weeks of capecitabine administration, followed by a one-week resting period. Pyridoxine (vitamin B6) was also administered for the prophylaxis of HFS.

**Figure 1:**
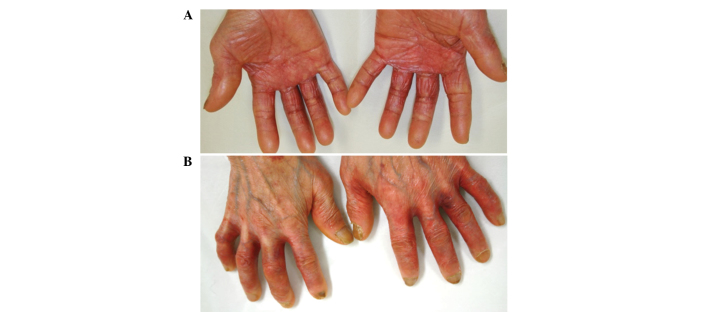
Captured images of the (A) palms and (B) backs of the hands, demonstrating grade 2 HFS. HFS, hand-foot syndrome.

**Figure 2:**
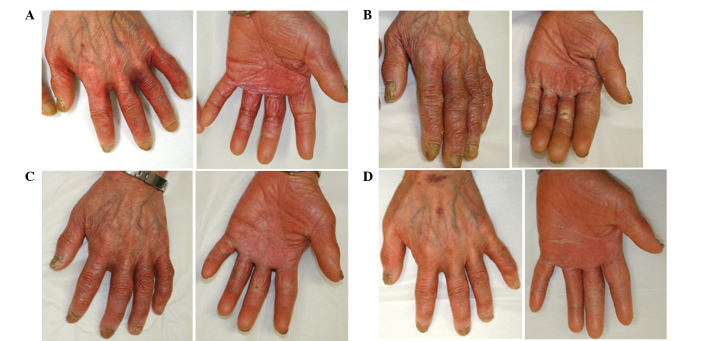
HFS due to chemotherapy with capecitabine. (A) Prior to adapalene gel application (grade 2). (B) One month (grade 1), (C) two months (grade 1) and (D) three months (grade 0) after adapalene gel application to the left hand. HFS, hand-foot syndrome.

Treatment with capecitabine was effective, however, following three courses, the patient complained of painful erythema of the palms, fingers and soles of the feet. Furthermore, the patient stated that the skin on the fingers and soles had peeled off and that the underlying dermis felt extremely stiff and tight. The patient was diagnosed with grade 3 HFS and was instructed to apply clobetasol propionate (a superpotent steroid) to the hands and feet following the cessation of capecitabine treatment. The grade 3 HFS improved to grade 2, however, the areas remained painful and the skin lesions continued to desquamate (Fig. 1). The patient was recommended to discontinue the chemotherapy until they had recovered from HFS, but the individual did not wish to discontinue capecitabine due to cancer recurrence. Therefore, the application of 0.1% adapalene gel was initiated to the affected areas on the hands twice daily. One month later, the inflammation of the individual’s hands gradually improved and the patient continued with the next course of capecitabine. The application of adapalene was continued and a marked reduction in HFS was observed, which resulted the reduction of inflammation and pain to grade 0 within 3 months (Fig. 2). The topical steroid was discontinued. The patient has remained in complete remission since pleural recurrence 2 years previously.

## Discussion

The current report presents a case with the successful treatment of refractory HFS induced by capecitabine using the topical application of adapalene. We hypothesized that topical adapalene induced the local chemotherapeutic resistance of the skin in the present patient receiving capecitabine, consequently improving HFS.

There are various recommendations concerning topical approaches or systemic therapy for HFS. However, the evidence of their benefit is limited and controversial. With regard to systemic strategies, in certain studies, pyridoxine (10,11) has also been found to be beneficial as a therapy. In other previous studies, however, pyridoxine therapy has been shown to have no effect (12,13) and the precise mechanism of action remains unknown. Cyclooxygenase-2 inhibitors have also been shown to be effective as a systemic approach for the prophylaxis of chemotherapy-associated HFS in patients with stage II and III colorectal cancer (14). Local therapy using mild emollient creams or gels in patients with grade 1 HFS may prevent mechanical irritation of the skin on the palms and soles. Superpotent corticosteroids for blisters and erosions have been found to have a sufficient anti-inflammatory effect. By contrast, HFS may not be prevented using glucocorticoids (2). A sufficient effect has not been previously demonstrated with dimethylsulfoxide or with a urea/lactic-based topical keratolytic agent (15). Overall, the optimal therapy for the prevention and treatment of HFS has not yet been established. In the present case, topical clobetasol propionate and pyridoxine were administered to the patient, but were also found to be ineffective. The treatment for steroid-resistant HFS appears to be particularly difficult.

Adapalene is a third-generation synthetic retinoid, which contains a naphthoic acid backbone (7). In contrast to retinoic acid, adapalene exhibits selectivity for the nuclear retinoic acid receptor (RAR β/γ). Due to its receptor selectivity, it causes less skin irritation. Topical retinoids have shown beneficial efficacy and good safety profiles as therapeutic antiaging agents in the management of photodamaged skin. Of all the topical retinoid agents, only adapalene has been approved for acne treatment in Japan. Topical retinoids have been found to show the following clinical effects: i) Improvement of coarse wrinkling; ii) increased skin smoothness and decreased roughness; and iii) improvements to skin discoloration/dyschromia (16). One of the notable molecular mechanisms of retinoid action is the increase in HB-EGF expression in keratinocytes. HB-EGF is considered to be important for skin wound healing, by accelerating keratinocyte migration (9).

HB-EGF is a member of the epidermal growth factor family and is synthesized as a transmembrane protein that may undergo proteolytic cleavage at the cell surface to release a mature soluble 14–22-kDa N-terminal ectodomain (s-HB-EGF). This s-HB-EGF subsequently binds to and activates its receptors, EGF receptor (EGFR)/erbB1/HER1 or ErbB4/HER4. Following the binding of s-HB-EGF, the heterodimerization or homodimerization of these receptors drives signal transduction cascades, which have critical roles in diverse cell fates, including development, proliferation, differentiation and migration. EGFR activation leads to an intracellular signaling cascade via Ras activation, which stimulates the extracellular signal pathway-regulated kinase/mitogen-activated protein kinase pathway. Suganuma *et al* identified HB-EGF as a chemoresistance-related gene in gastric cancer by cDNA microarray (17). It was observed that HB-EGF was highly expressed in 5-FU and cisplatin-resistance groups. Therefore, HB-EGF may also induce resistance to capecitabine, which is metabolized to 5-FU. Chemotherapy induces an elevation in the expression of HB-EGF, which is largely dependent on the activation of chemoresistant genes, including activator protein-1 and nuclear factor-κB, indicating that chemotherapy-induced HB-EGF activation represents a critical mechanism for induced chemotherapeutic resistance (6). Thus, HB-EGF has emerged as a key molecule in the resistance to chemotherapeutic agents. These observations demonstrate that HB-EGF is not only a potent inducer of tumor growth, but also a predictor of response to chemotherapy. HB-EGF contributes to tumor aggressiveness by promoting invasion, metastasis and chemotherapeutic resistance. Chemotherapy treatments increase HB-EGF levels, and chemotherapy-induced HB-EGF activation may protect cells from chemotherapy-induced cell death. Similarly, we hypothesized that retinoid-induced HB-EGF may protect keratinocytes from chemotherapy. In other words, topical retinoids that increase HB-EGF expression are likely to induce local chemotherapeutic resistance in the skin of patients receiving chemotherapy and consequently, decrease the cutaneous side-effects of chemotherapy. Topical adapalene, which has an anti-inflammatory effect and increases HB-EGF in the keratinocyte, is predicted to be effective for the treatment of HFS.

Rittié *et al*(8) reported that topical retinoid treatment causes epidermal hyperplasia mediated by EGFR activation via specific induction of its ligand, HB-EGF. Therefore, the topical application of retinoids may be effective in controlling EGFR inhibitor-induced skin reactions. Specific case reports have indicated that acneiform eruption or periungual inflammation due to EGFR inhibitors have been reduced by topical adapalene (18,19). Lapatinib, a tyrosine kinase inhibitor of HER2 and EGFR, is effective in combination with capecitabine in females with HER2-positive metastatic breast cancer. According to a phase III trial, in females with HER2-positive advanced breast cancer who received lapatinib plus capecitabine or capecitabine alone, skin rashes occurred more often in the group who received combination therapy (20). A topical retinoid may be more effective for skin reactions caused by lapatinib plus capecitabine compared with capecitabine alone in patients with metastatic breast cancer.

In the present case, the HFS of the patient improved slowly over several months. We hypothesized that the topical retinoid does not directly repair the damaged epidermis, but may promote the skin cell turnover and result in the improvement of the cutaneous adverse reactions. It normally takes 14 days for post-mitotic epidermal cells to reach the stratum corneum (21) and therefore, the proliferation and differentiation of keratinocytes by adapalene is likely to result in the delayed appearance of skin improvement.

The most common and frequent adverse reaction of topical retinoids is known as the ‘retinoid reaction’, which is characterized by pruritus, a burning sensation at the application sites, erythema and peeling, but these usually present with minimal to mild intensity (22). In a series of previous comparative trials, adapalene has been shown to be significantly less irritating than various other retinoic acid formulations. Furthermore, adapalene has been shown to be safe in several previous studies (7). Due to its chemical structure, the absorption of adapalene through the human skin is low. However, avoidance of adapalene during early pregnancy is firmly recommended. By contrast, in specific reviews of EGFR inhibitor-induced skin reactions, the use of retinoids for skin rash has not been generally recommended due to the lack of comedones and the possible aggravation of xerosis and eczema (12,23,24). Adapalene is applied to facial acne, and as facial skin is more sensitive than the skin of the hands or feet, the adapalene-related skin problems of the hands or feet may be fewer than those of the facial skin. In the present case, the application of adapalene to the patient’s hands and feet did not produce any side-effects, such as pruritus, erythema or xerosis.

In conclusion, topical retinoids may be effective for the treatment of capecitabine-induced HFS, by increasing HB-EGF expression and decreasing cutaneous side-effects. However, the mechanism of action of this effect on HFS remains unclear. A pilot trial designed to evaluate the potential efficacy and toxicities of adapalene for preventing HFS is necessary. Such a trial is currently underway at Kanazawa University to demonstrate clinical efficacy. Further studies are required to establish the therapeutic effect of topical retinoids on HFS.
